# *N*′-(2-Hy­droxy-3-meth­oxy­benzyl­idene)pyrazine-2-carbohydrazide monohydrate

**DOI:** 10.1107/S2414314619017310

**Published:** 2020-01-10

**Authors:** Zhaodong Wang

**Affiliations:** a Chongqing Key Laboratory of Environmental, Materials & Remediation Technologies, Chongqing University of Arts and Sciences, Yongchuan, Chongqing, 402160, People’s Republic of China; University of Aberdeen, Scotland

**Keywords:** crystal structure, Schiff base, acyl­hydrazone ligand, hydrogen bonding

## Abstract

In the title hydrated Schiff base, C_13_H_12_N_4_O_3_·H_2_O, the dihedral angle between the aromatic rings is 5.06 (11)° and an intra­molecular O—H⋯N hydrogen bond closes an S(6) ring. In the crystal, O_w_—H⋯O and O_w_—H⋯N (w = water) hydrogen bonds link the components into centrosymmetric tetra­mers (two Schiff bases and two water mol­ecules). Longer N—H⋯O hydrogen bonds link the tetra­mers into [010] chains. A weak C—H⋯O hydrogen bond and aromatic π–π stacking between the pyrazine and phenyl rings [centroid–centroid separations = 3.604 (2) and 3.715 (2) Å] are also observed.

## Abstract 2

In the title hydrated Schiff base, the dihedral angle between the aromatic rings is 5.06 (11)° and an intra­molecular O—H⋯N hydrogen bond closes an S(6) ring. In the crystal, O_w_—H⋯O and O_w_—H⋯N (w = water) hydrogen bonds link the components into centrosymmetric tetra­mers (two Schiff bases and two water mol­ecules). Longer N—H⋯O hydrogen bonds link the tetra­mers into [010] chains.

## Graphical abstract

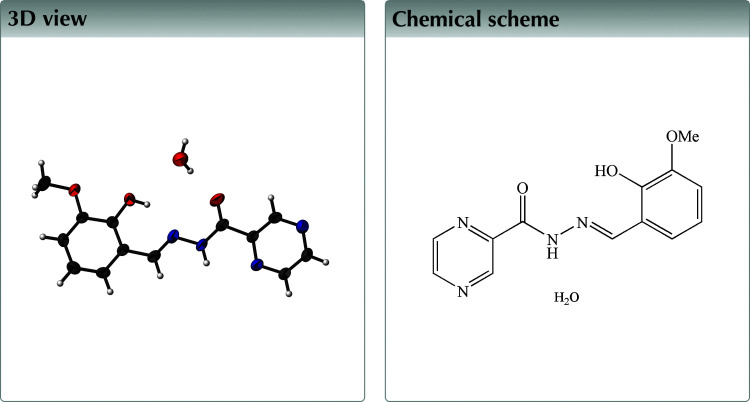

## Structure description

**Figure 1:**
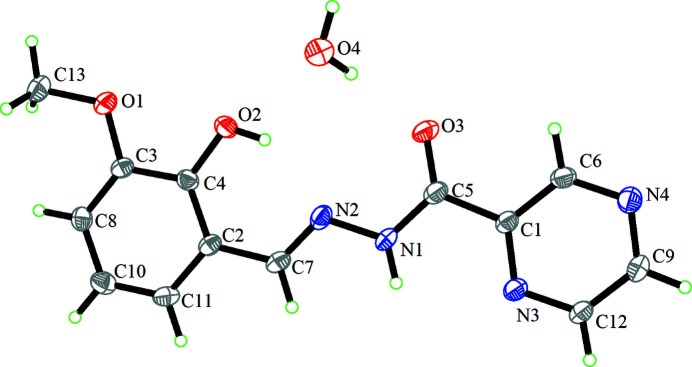
The mol­ecular structure of the title compound showing displacement ellipsoids drawn at the 30% probability level.

Hydrazone-type Schiff base ligands have attracted attention from inorganic chemists because of their simple synthesis and variety arising from changing the aldyhyde or ketone and acyl­hydrazide precursors. Their applications include mol­ecular switches (Coskun *et al.*, 2012 ▸), sensors (Albelda *et al.*, 2012 ▸) and single mol­ecular magnets (SMMs) (Anwar *et al.*, 2018 ▸). As part of our studies in this area, we now describe the synthesis and structure of the title pyrazine-containing hydrazone, which crystallized as a monohydrate (Fig. 1 ▸).

**Figure 2:**
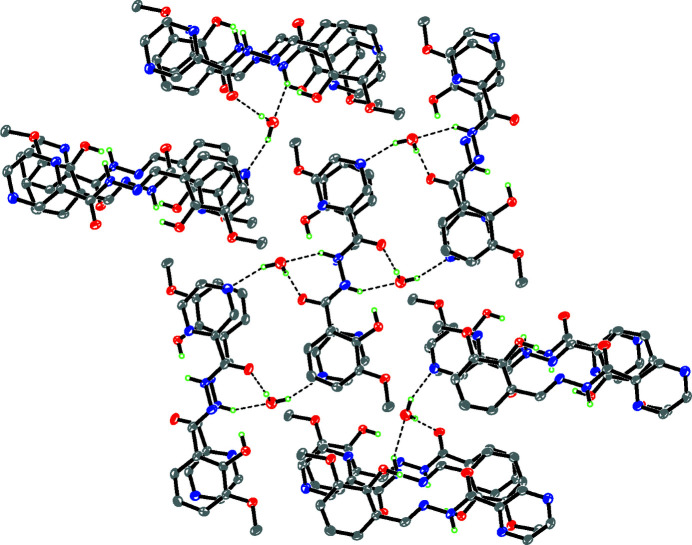
The crystal packing viewed along the -axis direction.

The dihedral angle between the aromatic rings is 5.06 (11)° and an intra­molecular O2—H2⋯N2 hydrogen bond closes an *S*(6) ring. The C7—N2 bond length [1.278 (3) Å] is consistent with a normal carbon–nitro­gen double bond. In the crystal, O_w_—H⋯O and O_w_—H⋯N (w = water) hydrogen bonds link the components into centrosymmetric tetra­mers (two Schiff base and two water mol­ecules). Longer N—H⋯O hydrogen bonds link the tetra­mers into [010] chains (Table 1 ▸, Fig. 2 ▸). The packing is consolidated by a weak C—H⋯O hydrogen bond and aromatic π–π stacking between the pyrazine and phenyl rings [centroid–centroid separations = 3.604 (2) and 3.715 (2) Å].

## Synthesis and crystallization

Pyrazine-2-carbohydrazide (2.76 g, 20 mmol) was reacted with 2-hy­droxy-3-meth­oxybenzaldehyde (3.04 g, 20 mmol) under reflux in 25 ml methanol for 8 h. After cooling and solvent removal by rotary evaporation, a light yellow solid was obtained, which was recrystallized from methanol solution at room temperature to obtain colourless crystals of the title compound.

## Refinement

Crystal data, data collection and structure refinement details are summarized in Table 2 ▸.

## Supplementary Material

Crystal structure: contains datablock(s) I. DOI: 10.1107/S2414314619017310/hb4335sup1.cif

Structure factors: contains datablock(s) I. DOI: 10.1107/S2414314619017310/hb4335Isup2.hkl

CCDC reference: 1973543

Additional supporting information:  crystallographic information; 3D view; checkCIF report

## Figures and Tables

**Table 1:** Hydrogen-bond geometry (Å, °)

*D*—H⋯*A*	*D*—H	H⋯*A*	*D*⋯*A*	*D*—H⋯*A*
N1—H1⋯N3	0.88	2.34	2.708 (3)	105
N1—H1⋯O4^i^	0.88	2.49	3.119 (3)	129
O2—H2⋯N2	0.84	1.94	2.668 (3)	145
O4—H4*A*⋯O3	0.87	1.99	2.846 (3)	167
O4—H4*B*⋯N4^ii^	0.87	2.17	2.998 (3)	160
C13—H13*A*⋯O2^iii^	0.98	2.56	3.335 (4)	135

Symmetry codes: (i) 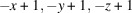 ; (ii) 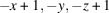 ; (iii) 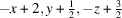 .

**Table 2:** Experimental details

Crystal data	
Chemical formula	C_13_H_12_N_4_O_3_·H_2_O
*M*_r_	290.28
Crystal system, space group	Monoclinic, *P*2_1_/*c*
Temperature (K)	189
*a*, *b*, *c* (Å)	7.018 (3), 9.041 (4), 20.828 (8)
β (°)	91.481 (7)
*V* (Å^3^)	1321.1 (9)
*Z*	4
Radiation type	Mo *K*α
μ (mm^−1^)	0.11
Crystal size (mm)	0.25 × 0.15 × 0.12

Data collection	
Diffractometer	Bruker D8 Venture
Absorption correction	Multi-scan (*SADABS*; Bruker, 2014 ▸)
*T*_min_, *T*_max_	0.626, 0.746
No. of measured, independent and observed [*I* > 2σ(*I*)] reflections	7707, 2996, 1745
*R*_int_	0.053
(sin θ/λ)_max_ (Å^−1^)	0.653

Refinement	
*R*[*F* ^2^ > 2σ(*F* ^2^)], *wR*(*F* ^2^), *S*	0.063, 0.161, 1.00
No. of reflections	2996
No. of parameters	195
H-atom treatment	H-atom parameters constrained
Δρ_max_, Δρ_min_ (e Å^−3^)	0.31, −0.28

Computer programs: *APEX2* and *SAINT* (Bruker, 2014 ▸), *SHELXT* (Sheldrick, 2015*a* ▸), *SHELXL* (Sheldrick, 2015*b* ▸) and *OLEX2* (Dolomanov *et al.*, 2009 ▸).

## full crystallographic data

### Crystal data

**Table:**

C_13_H_12_N_4_O_3_·H_2_O	*F*(000) = 608
*M**_r_* = 290.28	*D*_x_ = 1.459 Mg m^−^^3^
Monoclinic, *P*2_1_/*c*	Mo *K*α radiation, λ = 0.71073 Å
*a* = 7.018 (3) Å	Cell parameters from 1318 reflections
*b* = 9.041 (4) Å	θ = 2.5–24.4°
*c* = 20.828 (8) Å	µ = 0.11 mm^−^^1^
β = 91.481 (7)°	*T* = 189 K
*V* = 1321.1 (9) Å^3^	Block, colourless
*Z* = 4	0.25 × 0.15 × 0.12 mm

### Data collection

**Table:**

Bruker D8 Venture diffractometer	1745 reflections with *I* > 2σ(*I*)
Multi–scan	*R*_int_ = 0.053
Absorption correction: multi-scan (SADABS; Bruker, 2014)	θ_max_ = 27.7°, θ_min_ = 2.5°
*T*_min_ = 0.626, *T*_max_ = 0.746	*h* = −9→8
7707 measured reflections	*k* = −11→11
2996 independent reflections	*l* = −18→27

### Refinement

**Table:**

Refinement on *F*^2^	Primary atom site location: dual
Least-squares matrix: full	Hydrogen site location: mixed
*R*[*F*^2^ > 2σ(*F*^2^)] = 0.063	H-atom parameters constrained
*wR*(*F*^2^) = 0.161	*w* = 1/[σ^2^(*F*_o_^2^) + (0.076*P*)^2^] where *P* = (*F*_o_^2^ + 2*F*_c_^2^)/3
*S* = 1.00	(Δ/σ)_max_ < 0.001
2996 reflections	Δρ_max_ = 0.31 e Å^−^^3^
195 parameters	Δρ_min_ = −0.28 e Å^−^^3^
0 restraints	

### Special details

**Table:**

Geometry. All esds (except the esd in the dihedral angle between two l.s. planes) are estimated using the full covariance matrix. The cell esds are taken into account individually in the estimation of esds in distances, angles and torsion angles; correlations between esds in cell parameters are only used when they are defined by crystal symmetry. An approximate (isotropic) treatment of cell esds is used for estimating esds involving l.s. planes.

### Fractional atomic coordinates and isotropic or equivalent isotropic displacement parameters (Å^2^)

**Table:**

	*x*	*y*	*z*	*U*_iso_*/*U*_eq_	
O1	0.8752 (3)	0.71932 (19)	0.73561 (7)	0.0466 (5)	
O2	0.8069 (3)	0.55020 (18)	0.63673 (8)	0.0485 (5)	
H2	0.784669	0.504827	0.602149	0.073*	
O3	0.6841 (3)	0.2318 (2)	0.50428 (8)	0.0551 (6)	
N1	0.7156 (3)	0.4515 (2)	0.45331 (9)	0.0390 (5)	
H1	0.713885	0.499088	0.416450	0.047*	
N2	0.7502 (3)	0.5261 (2)	0.51003 (9)	0.0380 (5)	
N3	0.6642 (3)	0.3166 (2)	0.33806 (9)	0.0366 (5)	
N4	0.5903 (3)	0.0122 (2)	0.33186 (10)	0.0411 (5)	
C1	0.6523 (3)	0.2336 (3)	0.39063 (10)	0.0324 (6)	
C2	0.8247 (3)	0.7542 (3)	0.56268 (10)	0.0329 (6)	
C3	0.8709 (3)	0.7883 (3)	0.67721 (11)	0.0325 (6)	
C4	0.8336 (3)	0.6947 (3)	0.62434 (11)	0.0336 (6)	
C5	0.6843 (3)	0.3044 (3)	0.45503 (11)	0.0370 (6)	
C6	0.6124 (3)	0.0832 (3)	0.38743 (11)	0.0378 (6)	
H6	0.600418	0.029255	0.426269	0.045*	
C7	0.7873 (3)	0.6640 (3)	0.50590 (11)	0.0368 (6)	
H7	0.790496	0.708891	0.464695	0.044*	
C8	0.8975 (3)	0.9374 (3)	0.66768 (12)	0.0388 (6)	
H8	0.924031	1.000252	0.703370	0.047*	
C9	0.6062 (4)	0.0945 (3)	0.27911 (12)	0.0400 (6)	
H9	0.594317	0.048417	0.238199	0.048*	
C10	0.8859 (4)	0.9966 (3)	0.60621 (13)	0.0431 (7)	
H10	0.902978	1.099855	0.600130	0.052*	
C11	0.8502 (3)	0.9074 (3)	0.55447 (12)	0.0395 (6)	
H11	0.842440	0.948922	0.512593	0.047*	
C12	0.6397 (3)	0.2452 (3)	0.28243 (11)	0.0387 (6)	
H12	0.645434	0.299940	0.243586	0.046*	
C13	0.8953 (4)	0.8115 (3)	0.79116 (11)	0.0505 (7)	
H13A	1.018576	0.862348	0.790623	0.076*	
H13B	0.888839	0.750474	0.829937	0.076*	
H13C	0.792400	0.884721	0.790985	0.076*	
O4	0.4937 (3)	0.2931 (2)	0.62032 (9)	0.0573 (6)	
H4A	0.567749	0.271798	0.588742	0.086*	
H4B	0.487099	0.211456	0.642269	0.086*	

### Atomic displacement parameters (Å^2^)

**Table:**

	*U*^11^	*U*^22^	*U*^33^	*U*^12^	*U*^13^	*U*^23^
O1	0.0713 (13)	0.0426 (11)	0.0256 (9)	−0.0046 (9)	−0.0038 (8)	−0.0012 (8)
O2	0.0798 (14)	0.0288 (10)	0.0360 (10)	−0.0038 (9)	−0.0165 (9)	0.0014 (8)
O3	0.0919 (15)	0.0470 (12)	0.0263 (10)	0.0178 (10)	−0.0010 (9)	0.0063 (8)
N1	0.0536 (14)	0.0429 (13)	0.0202 (10)	0.0006 (10)	−0.0043 (9)	−0.0019 (9)
N2	0.0425 (12)	0.0447 (14)	0.0265 (11)	0.0051 (10)	−0.0051 (9)	−0.0052 (9)
N3	0.0413 (12)	0.0401 (12)	0.0283 (11)	0.0018 (9)	−0.0009 (9)	0.0017 (9)
N4	0.0436 (13)	0.0414 (13)	0.0383 (13)	0.0000 (10)	−0.0011 (10)	−0.0032 (10)
C1	0.0333 (13)	0.0379 (14)	0.0259 (12)	0.0062 (10)	−0.0013 (9)	0.0001 (11)
C2	0.0319 (13)	0.0409 (15)	0.0258 (12)	0.0006 (11)	−0.0034 (10)	0.0012 (11)
C3	0.0318 (13)	0.0385 (15)	0.0270 (12)	0.0007 (10)	−0.0024 (10)	0.0033 (10)
C4	0.0363 (14)	0.0288 (14)	0.0353 (14)	0.0023 (10)	−0.0039 (10)	0.0025 (10)
C5	0.0418 (15)	0.0433 (16)	0.0258 (13)	0.0101 (11)	0.0002 (11)	0.0014 (11)
C6	0.0452 (15)	0.0388 (15)	0.0294 (13)	0.0062 (11)	0.0013 (11)	0.0063 (11)
C7	0.0371 (14)	0.0472 (17)	0.0257 (13)	0.0019 (11)	−0.0039 (10)	0.0036 (11)
C8	0.0439 (15)	0.0346 (15)	0.0376 (14)	−0.0042 (11)	−0.0034 (11)	−0.0059 (11)
C9	0.0428 (14)	0.0477 (17)	0.0294 (13)	−0.0024 (12)	−0.0011 (11)	−0.0078 (12)
C10	0.0449 (15)	0.0361 (15)	0.0482 (16)	−0.0040 (12)	−0.0038 (12)	0.0050 (12)
C11	0.0418 (14)	0.0422 (16)	0.0341 (14)	−0.0046 (12)	−0.0044 (11)	0.0115 (11)
C12	0.0462 (15)	0.0438 (16)	0.0258 (13)	−0.0019 (12)	−0.0037 (11)	0.0014 (11)
C13	0.0620 (19)	0.0581 (19)	0.0314 (14)	0.0005 (14)	−0.0002 (13)	−0.0079 (13)
O4	0.0784 (15)	0.0496 (12)	0.0441 (12)	0.0017 (10)	0.0057 (10)	0.0024 (9)

### Geometric parameters (Å, º)

**Table:**

O1—C3	1.367 (3)	C3—C4	1.408 (3)
O1—C13	1.430 (3)	C3—C8	1.376 (3)
O2—H2	0.8400	C6—H6	0.9500
O2—C4	1.346 (3)	C7—H7	0.9500
O3—C5	1.218 (3)	C8—H8	0.9500
N1—H1	0.8800	C8—C10	1.388 (4)
N1—N2	1.376 (3)	C9—H9	0.9500
N1—C5	1.348 (3)	C9—C12	1.385 (4)
N2—C7	1.278 (3)	C10—H10	0.9500
N3—C1	1.332 (3)	C10—C11	1.364 (4)
N3—C12	1.333 (3)	C11—H11	0.9500
N4—C6	1.329 (3)	C12—H12	0.9500
N4—C9	1.334 (3)	C13—H13A	0.9800
C1—C5	1.498 (3)	C13—H13B	0.9800
C1—C6	1.390 (3)	C13—H13C	0.9800
C2—C4	1.393 (3)	O4—H4A	0.8702
C2—C7	1.455 (3)	O4—H4B	0.8697
C2—C11	1.407 (3)		
			
C3—O1—C13	117.0 (2)	N2—C7—C2	121.7 (2)
C4—O2—H2	109.5	N2—C7—H7	119.1
N2—N1—H1	120.5	C2—C7—H7	119.1
C5—N1—H1	120.5	C3—C8—H8	119.8
C5—N1—N2	119.1 (2)	C3—C8—C10	120.4 (2)
C7—N2—N1	116.9 (2)	C10—C8—H8	119.8
C1—N3—C12	115.6 (2)	N4—C9—H9	119.2
C6—N4—C9	116.0 (2)	N4—C9—C12	121.7 (2)
N3—C1—C5	119.0 (2)	C12—C9—H9	119.2
N3—C1—C6	121.9 (2)	C8—C10—H10	119.8
C6—C1—C5	119.1 (2)	C11—C10—C8	120.4 (2)
C4—C2—C7	122.4 (2)	C11—C10—H10	119.8
C4—C2—C11	119.3 (2)	C2—C11—H11	119.8
C11—C2—C7	118.3 (2)	C10—C11—C2	120.5 (2)
O1—C3—C4	114.9 (2)	C10—C11—H11	119.8
O1—C3—C8	125.2 (2)	N3—C12—C9	122.5 (2)
C8—C3—C4	120.0 (2)	N3—C12—H12	118.7
O2—C4—C2	123.3 (2)	C9—C12—H12	118.7
O2—C4—C3	117.2 (2)	O1—C13—H13A	109.5
C2—C4—C3	119.4 (2)	O1—C13—H13B	109.5
O3—C5—N1	123.9 (2)	O1—C13—H13C	109.5
O3—C5—C1	121.4 (2)	H13A—C13—H13B	109.5
N1—C5—C1	114.7 (2)	H13A—C13—H13C	109.5
N4—C6—C1	122.2 (2)	H13B—C13—H13C	109.5
N4—C6—H6	118.9	H4A—O4—H4B	104.5
C1—C6—H6	118.9		
			
O1—C3—C4—O2	−0.1 (3)	C6—N4—C9—C12	−1.3 (4)
O1—C3—C4—C2	179.5 (2)	C6—C1—C5—O3	3.2 (4)
O1—C3—C8—C10	−178.5 (2)	C6—C1—C5—N1	−177.8 (2)
N1—N2—C7—C2	179.5 (2)	C7—C2—C4—O2	−0.6 (4)
N2—N1—C5—O3	0.2 (4)	C7—C2—C4—C3	179.8 (2)
N2—N1—C5—C1	−178.74 (19)	C7—C2—C11—C10	−179.9 (2)
N3—C1—C5—O3	−176.4 (2)	C8—C3—C4—O2	−179.3 (2)
N3—C1—C5—N1	2.6 (3)	C8—C3—C4—C2	0.2 (3)
N3—C1—C6—N4	2.4 (4)	C8—C10—C11—C2	0.0 (4)
N4—C9—C12—N3	2.2 (4)	C9—N4—C6—C1	−0.9 (4)
C1—N3—C12—C9	−0.7 (3)	C11—C2—C4—O2	178.5 (2)
C3—C8—C10—C11	−0.8 (4)	C11—C2—C4—C3	−1.0 (3)
C4—C2—C7—N2	3.9 (4)	C11—C2—C7—N2	−175.3 (2)
C4—C2—C11—C10	0.9 (4)	C12—N3—C1—C5	178.1 (2)
C4—C3—C8—C10	0.7 (4)	C12—N3—C1—C6	−1.5 (3)
C5—N1—N2—C7	177.0 (2)	C13—O1—C3—C4	−174.5 (2)
C5—C1—C6—N4	−177.1 (2)	C13—O1—C3—C8	4.7 (3)

### Hydrogen-bond geometry (Å, º)

**Table:**

*D*—H···*A*	*D*—H	H···*A*	*D*···*A*	*D*—H···*A*
N1—H1···N3	0.88	2.34	2.708 (3)	105
N1—H1···O4^i^	0.88	2.49	3.119 (3)	129
O2—H2···N2	0.84	1.94	2.668 (3)	145
O4—H4*A*···O3	0.87	1.99	2.846 (3)	167
O4—H4*B*···N4^ii^	0.87	2.17	2.998 (3)	160
C13—H13*A*···O2^iii^	0.98	2.56	3.335 (4)	135

Symmetry codes: (i) −*x*+1, −*y*+1, −*z*+1; (ii) −*x*+1, −*y*, −*z*+1; (iii) −*x*+2, *y*+1/2, −*z*+3/2.
